# STAT3 Mediates the Differential Effects of Oncostatin M and TNFα on RA Synovial Fibroblast and Endothelial Cell Function

**DOI:** 10.3389/fimmu.2019.02056

**Published:** 2019-08-28

**Authors:** Megan M. Hanlon, Tatsiana Rakovich, Clare C. Cunningham, Sharon Ansboro, Douglas J. Veale, Ursula Fearon, Trudy McGarry

**Affiliations:** ^1^Molecular Rheumatology, Trinity Biomedical Sciences Institute, TCD, Dublin, Ireland; ^2^Centre for Arthritis and Rheumatic Diseases, St. Vincent's University Hospital, UCD, Dublin, Ireland

**Keywords:** rheumatoid arthritis, cellular bioenergetics, pro-inflammatory cytokines, JAK-STAT signaling, synovial fibroblasts

## Abstract

**Objectives:** Oncostatin M (OSM), a pleiotropic cytokine and a member of the gp130/IL-6 cytokine family, has been implicated in the pathogenesis of autoimmune diseases. Here we investigate the mechanisms by which its synergistic interactions with TNFα regulate the cellular bioenergetics and invasive function of synovial cells from patients with Rheumatoid Arthritis.

**Methods:** Primary RA synovial fibroblasts (RAFLS) and human umbilical vein endothelial cells (HUVEC) were cultured with OSM alone or in combination with TNFα. Pro-inflammatory cytokines, angiogenic growth factors and adhesion molecules were quantified by real-time PCR and ELISA. Invasion, angiogenesis and cellular adhesion were quantified by Transwell invasion chambers, Matrigel tube formation assays, and adhesion binding assays. Cellular bioenergetics was assessed using the Seahorse XFe96 Analyser. Key metabolic genes (GLUT-1, HK2, PFKFB3, HIF1α, LDHA, PKM2) and transcription factor STAT3 were measured using real-time PCR and western blot.

**Results:** OSM differentially regulates pro-inflammatory mediators in RAFLS and HUVEC, with IL-6, MCP-1, ICAM-1, and VEGF all significantly induced, in contrast to the observed inhibition of IL-8 and GROα, with opposing effects observed for VCAM-1 depending on cell type. Functionally, OSM significantly induced angiogenic network formation, adhesion, and invasive mechanisms. This was accompanied by a change in the cellular bioenergetic profile of the cells, where OSM significantly increased the ECAR/OCR ratio in favor of glycolysis, paralleled by induction of the glucose transporter GLUT-1 and key glycolytic enzymes (HK2, PFKFB3, HIF1α). OSM synergizes with TNFα to differentially regulate pro-inflammatory mechanisms in RAFLS and HUVEC. Interestingly, OSM differentially synergizes with TNFα to regulate metabolic reprogramming, where induction of glycolytic activity with concomitant attenuation of mitochondrial respiration and ATP activity was demonstrated in RAFLS but not in HUVEC. Finally, we identified a mechanism, whereby the combination of OSM with TNFα induces transcriptional activity of STAT3 only in RAFLS, with no effect observed in HUVEC.

**Conclusion:** STAT3 mediates the differential effects of OSM and TNFα on RAFLS and EC function. Targeting OSM or downstream signaling pathways may lead to new potential therapeutic or adjuvant strategies, particularly for those patients who have sub-optimal responses to TNFi.

## Introduction

Rheumatoid arthritis (RA) is a chronic autoimmune disease characterized by synovial hyperplasia and degradation of articular cartilage and bone, ultimately leading to irreversible disability. Although the initiating trigger for RA is not known, angiogenesis is one of the earliest events in the pathogenesis of this disease. Sprouting angiogenesis allows for a self-perpetuating influx of immune cells into the synovial joint, resulting in expansion of the synovial tissue into an aggressive tumor-like pannus (1). Despite this increased vascular supply, studies have demonstrated that the synovial joint is profoundly hypoxic (2). This is due to the highly dysfunctional and immature nature of the vasculature resulting in abnormal blood flow supplying inadequate nutrients and oxygen to the expanding synovium. Thus, the increasing metabolic turnover of the pannus outpaces vascular supply, rendering the inflamed synovium hypoxic (3–6).

The importance of metabolism in regulating synovial inflammation has recently emerged with many studies indicating that immune and stromal cells undergo a bioenergetic switch to a highly metabolically active state in order to meet the energy demands of the expanding synovium (7, 8). Indeed, the metabolic milieu of the inflamed joint reflects the chronically active state of immune and stromal cells, with elevated lactate levels and reduced glucose observed in RA synovial fluid, along with increased glycolytic enzyme activity and accumulation of succinate in synovial fluid and tissue (9–13). Recent studies have shown that treatment with glycolytic inhibitors dampens cytokine production, invasive mechanisms, and key transcriptional regulators in various synovial cells while also improving disease severity in animal models of arthritis (9, 14, 15).

The cytokine Oncostatin M (OSM) is highly expressed in the RA joint, and shares a common receptor signal subunit (gp-130) with IL-6-type cytokines (16). Produced mainly by macrophages, neutrophils and activated T-cells, OSM signals via the Janus Kinase (JAK) family of receptor-associated tyrosine kinases and is associated with the activation of STAT3 (17–19). Increased expression of OSM is associated with a plethora of pathologies including atherosclerosis, psoriasis, and many cancers (20, 21). Most recently, OSM has been shown to play a role in inflammatory bowel disease (IBD) with a study demonstrating heightened expression of OSM and its receptor in the inflamed IBD intestine, correlating with disease severity (22).

In the context of RA, overexpression of OSM in synovial fluid and tissue has been observed with levels correlating with joint inflammation (23). Collectively, studies have demonstrated that OSM plays a critical role in RAFLS activation, promotion of angiogenesis, adhesion molecules and chemokines from RAFLS, altering the matrix metalloproteinases (MMP)/tissue inhibitor of matrix metalloproteinases (TIMP) ratio and inducing RANKL in RAFLS and chondrocytes in favor of joint destruction (21, 24, 25). Blocking OSM in a collagen-induced arthritis mouse model improves joint inflammation and cartilage damage (26). Furthermore, recent studies have demonstrated that inhibition of OSM-induced RAFLS pro-inflammatory mechanisms and cartilage degradation are rescued in the presence of JAK-STAT inhibitors (27, 28), effects that are, in part, mediated by a switch in the metabolic profile of the cell (29). Conversely, OSM is a pleiotropic cytokine often displaying divergent effects with both pro- and anti-inflammatory effects depending on the cell type and microenvironment. Previous studies have shown that OSM can inhibit IL-1-induced IL-8 and granulocyte macrophage colony stimulating factor (GM-CSF) and promote TIMP expression in RAFLS (24, 30). OSM inhibits TNFα- and IL-17A-induced TIMP-1 while potentiating IL-1β-induced TIMP-1 expression in RAFLS (24, 31). More recently, studies have demonstrated that OSM can inhibit Th17 differentiation in mouse models of arthritis through reciprocal regulation of SOCS3, STAT3, and STAT5 (32). Therefore, the role of OSM in RA disease pathology is complex, depending largely on cell type and microenvironment.

Given the pivotal role of metabolism in regulating synovial inflammation, in this study we examined the effect of OSM on pro-inflammatory, angiogenic, and bioenergetic mechanisms in RAFLS and HUVEC. Furthermore, we investigated the relationship between OSM and the major pro-inflammatory cytokine; tumor necrosis factor α (TNFα), a central player in inflammation and destruction in the RA joint.

## Materials and Methods

### Patient Recruitment and Arthroscopy

RA patients were recruited from the Rheumatology Department, St. Vincent's University Hospital. All patients gave fully informed written consent approved by the St. Vincent's University Hospital, Ethics and Medical Research Committee and research was performed in accordance with the Declaration of Helsinki. Synovial tissue biopsies were obtained at arthroscopy under local anesthetic using a Wolf 2.7 mm telescope (Wolf—Germany) as previously described (2). Biopsies were utilized for isolation of primary RA synovial fibroblasts (RAFLS).

### Isolation of Primary Fibroblasts

RA synovial biopsies were digested with 1 mg/ml collagenase type 1 (Worthington Biochemical, Freehold, NJ, USA) in RPMI-1640 (Gibco-BRL, Paisley, UK) for 4 h at 37°C in humidified air with 5% CO_2_. Dissociated cells were grown to confluence in RPMI 1640, 10% FCS (Gibco-BRL), 10 ml of 1 mmol/l HEPES (Gibco-BRL), penicillin (100 units/ml; Bioscience), streptomycin (100 units/ml; Bioscience) and fungizone (0.25 μg/ml; Bioscience) before passaging. Cells were used between passages 3–8.

### Culture of HUVEC

Human umbilical vein endothelial cells (HUVEC) (ATCC, Manassas, USA) were grown to confluence in endothelial cell basal media (MCDB-131, Gibco) supplemented with L-Glutamine (20 ml of 100X solution), Hydrocortisone (0.6 μg/ml), hEGF (0.01 μg/ml), Penicillin/Streptomycin (100 units/ml; Biosciences), Fungizone (0.25 μg/ml; Biosciences) and 15% FCS (Gibco-BRL).

### Cytokine and Chemokine Measurements

To assess the effects of OSM on pro-inflammatory mediators, RAFLS/HUVEC were seeded in 48-well plates at a density of 3 × 10^5^ and allowed to attach overnight. Cells were incubated in serum-free RPMI-1640 or MCDB-131 for 24 h and subsequently stimulated with OSM (10 ng/ml). For synergy experiments, cells were also incubated in the presence or absence of TNFα (0.01, 0.1, 1 ng/ml). Supernatants were harvested and levels of IL-6, MCP-1, IL-8, RANTES, and GROα were measured by specific ELISA (MCP-1: eBiosciences, USA, IL-6, IL-8, RANTES, GROα; R&D systems, UK) according to manufacturer's conditions.

### Transwell Invasion Assay

Biocoat Matrigel™ Invasion Chambers (Becton Dickinson, UK) were used to assess RAFLS/HUVEC invasion. Cells were seeded at a density of either 3.5 × 10^4^ (RAFLS) or 2.5 × 10^4^ (HUVEC) cells per well in the migration chamber on 8 μm membranes pre-coated with matrigel. Cells were incubated with OSM (10 ng/ml) for 24 h (HUVEC) or 48 h (RAFLS). Non-migrating cells were removed from the upper surface by gentle scrubbing. Migrating cells attached to the lower membrane were fixed with 4% paraformaldehyde and stained with 0.1% crystal violet. Cells from five random high power fields for each well were counted to assess the average number of invading cells.

### HUVEC Tube Formation

Matrigel (50 μl; BD Biosciences, San Jose, CA, USA) was plated in 96-well culture plates after thawing on ice and allowed to polymerise for 30 min at 37°C in humidified air with 5% CO_2_. 2 × 10^4^ cells in supplemented MCDB-131 was added to each well and cells were stimulated with OSM (10 ng/ml) on control medium for 24 h. EC tubule formation was then assessed using phase-contrast microscopy. Cells were quantified by counting the number of connecting branches formed from five random high power fields as previously described (24).

### Adhesion Assay

RAFLS/HUVEC were grown to confluence in 24-well plates, incubated in serum-free RPMI-1640 or MCDB-131 for 24 h and subsequently stimulated with OSM (10 ng/ml) for a further 24 h. PBMC from healthy donors were isolated by density gradient centrifugation (Lymphoprep; Stemcell Technologies) according to the manufacturer's recommendations. PBMC were then re-suspended in MCDB-131. 7.5 × 10^4^ PBMC were added to each well containing RAFLS/HUVEC and incubated at 37°C with 5% CO_2_ for 1 h. After the incubation time, supernatants were removed and wells were washed with PBS. Semi-quantification was performed by counting adherent PBMCs as viewed under phase-contrast microscopy (Leica, Germany) at 10 × magnification. Cells from five random high power fields for each well were counted to assess the average number of adherent cells.

### mRNA Extraction and cDNA Synthesis

To assess the effects of OSM on specific genes, RAFLS/HUVEC were seeded into 6-well plates and allowed to grow to confluence. Cells were incubated in serum-free RPMI-1640 or MCDB-131 for 24 h and subsequently stimulated with OSM (10 ng/ml). For synergy experiments, cells were also incubated in the presence or absence of TNFα (1 ng/ml). Total RNA was isolated using the miRNeasy Mini Kit (Qiagen, Germany) according to the manufacturer's protocol. The integrity of RNA samples were assessed using a bioanalyzer (Agilent, CA, USA). Samples with a 260:280 nm ratio of 1.8 and above and an RNA integrity number between 7 and 10 were used in subsequent experiments. Isolated RNA was stored at −80°C. Total RNA (100 ng) was reverse transcribed to cDNA using a high capacity cDNA reverse transcription kit (Applied Biosystems, Cheshire, UK) and stored at −20°C until further use.

### RT-PCR Analysis

Gene expression data were quantified by RT-PCR using the Quant Studio 5 Thermal Cycler (Applied Biosystem, Lewes, UK). Reaction mixtures contained 1 μl of cDNA, SYBR green I PCR mastermix (Applied Biosystems) and target mRNA specific primer pairs as follows: VEGF for 5′ GCAGAATCATCACGAAGTGGTG 3′ VEGF rev 5′ TCTCGATTGGATGGCAGTAGCT 3′, VCAM-1 for 5′ GTAAAAGAATTGCAA GTC TACATATCAC 3′, VCAM-1 rev 5′ GATGGATTCACAGAAATAACTGTATTC 3′, ICAM-1 for 5′ AACCAGAGCCAGGAGACACTG 3′, ICAM-1 rev 5′ GCGCCGGAAAGCTGTAGATG 3′, HIF1α for 5′ GAAACTTCTGGATGCTGGTGATTT 3′, HIF1α rev 5′ GCAATTCATCTGTGCTTTCATGTCA 3′, HK2 for 5′ TTCTTGTCTCAG ATTGAGAGTGAC 3′, HK2 rev 5′ TTGCAGGATGGCTCGGACTTG 3′, LDHA for 5′ ATGGAGATTCCAGTGTGCCTGT 3′, LDHA rev 5′ CAGAGAGACACCAGCAACATTC 3′, GLUT1 for 5′ CTTCCAGTATGTGGAGCAACTGT, GLUT1 rev 5′ GCACAGTGAAGATGATGAAGACG 3′, PFKFB3 for 5′ ACCAAAGATCACCCACGGATGT 3′, PFKFB3 rev 5′ AGCGAGTGCAGAATGGACACAA 3′, PKM2 for 5′ ATTATTTGAGGAACTCCGCCG 3′, PKM2 rev 5′ ATTCCGGGTCACAGCAATGAT 3′, STAT3 for 5′ TTCACTTGGGTGGAGAAG 3′ and STAT3 rev 5′ CGGACTGGATCTGGGTCT 3′. Samples lacking multiscribe reverse transcriptase formed negative controls to ensure target-specific quantification. Data were analyzed using the comparative threshold cycle (Ct) method with normalization to the expression of RPLPO (for 5′ GCGTCCTCGTGGAAGTGACATCG 3′, rev 5′ TCAGGGATTGCCACGCAGGG 3′) and HPRT1 (for 5′ ATGGACAGGACTGAACGTCTTG 3′, rev 5′ GGCTACAATGTGATGGCCTC 3′) as endogenous controls.

### Cellular Bioenergetic Function Analysis

Oxygen consumption rate (OCR) and extracellular acidification rate (ECAR), reflecting oxidative phosphorylation and glycolysis, respectively, were measured using the Seahorse-XFe96 analyzer (Seahorse Biosciences). RAFLS/HUVEC were seeded at 15,000 cells per well in a 96-well cell culture XFe microplate (Seahorse Biosciences) and allowed to adhere overnight. Following this, cells were then cultured with OSM (10 ng/ml) for 3 h. For synergy experiments, cells were also incubated in the presence or absence of TNFα (1 ng/ml) for 3 h. Cells were then washed with assay medium (unbuffered DMEM supplemented with 10 mM glucose, pH-7.4) before incubation with assay medium for 30 min at 37°C in a non-CO_2_ incubator. Basal oxidative phosphorylation/glycolysis was calculated by the average of three baseline OCR/ECAR measurements, respectively, obtained before injection of specific metabolic inhibitors; oligomycin (ATP-synthase-inhibitor) (2 μg/ml; Seahorse Biosciences, UK) trifluorocarbonylcyanide phenylhydrazone (FCCP) (mitochondrial uncoupler) (5 μM; Seahorse Biosciences) and antimycin A (complex-III inhibitor) (2 μM; Seahorse Biosciences). Oligomycin was injected to evaluate both the maximal glycolytic rate and ATP synthesis, determined by subtracting the amount of respiration left after oligomycin injection from baseline OCR. FCCP was injected to evaluate the maximal respiratory capacity (average of three measurements following injection). Maximal respiratory capacity was determined by subtracting baseline OCR from FCCP-induced OCR and the respiratory reserve (baseline OCR subtracted from maximal respiratory capacity).

### Protein Isolation and Western Blotting Analysis

RAFLS/HUVEC were grown to confluence in 6-well plates. Once confluent, cells were incubated in serum-free RPMI-1640 or MCDB-131 for 24 h and subsequently stimulated with OSM (10 ng/ml). For synergy experiments, cells were also incubated in the presence or absence of TNFα (1 ng/ml). Cells were trypsinized and collected prior to cell lysis. Ice-cold RIPA (Radio-Immunoprecipitation Assay) buffer (Sigma) containing 10 μg/ml phosphatase inhibitor cocktail and 10 μg/ml protease inhibitor cocktail (Sigma) was used to extract protein from HUVEC/RAFLS. Measurement of protein concentration was performed using a BCA assay (Pierce Chemical Co, Rockford, IL, USA). Protein (2–5 μg) was resolved by SDS-PAGE (5% stacking, 10% resolving), gels were then transferred onto nitrocellulose membranes (Amersham Biosciences, Buckinghamshire, UK) prior to 1 h blocking in wash buffer containing 5% non-fat milk with gentle agitation at room temperature. Membranes were incubated with mouse monoclonal anti-HK2 (Novus Biologicals, USA), rabbit monoclonal anti-PFKFB3 (Abcam, UK), rabbit polyclonal anti-GLUT-1 (Abcam), anti-pSTAT3, and anti-total STAT3 (Cell-Signaling Technology, UK) diluted in 5% non-fat milk containing 0.1% Tween 20 at 4°C overnight with gentle agitation. β-actin (1:5,000, Sigma) was used as a loading control. Following three 15 min washes, membranes were incubated with appropriate horseradish peroxidase-conjugated secondary antibodies (1:5,000) for 3 h at room temperature. The signal was detected using SuperSignal® West Pico Chemiluminescent Substrate (Amersham Biosciences). Band densities were imaged using the ChemiDoc MP Imaging System (Bio-Rad, USA).

### Statistical Analysis

Statistical analyses were performed using Prism 5 software. Wilcoxon Signed Rank test or Mann Whitney was used for analysis of non-parametric data. Student *t*-test was used for parametric data. *P*-values of < 0.05 (^*^p < 0.05) were determined as statistically significant.

## Results

### OSM Differentially Regulates Cytokine and Chemokine Secretion in RAFLS and HUVEC

**Figure 1:**
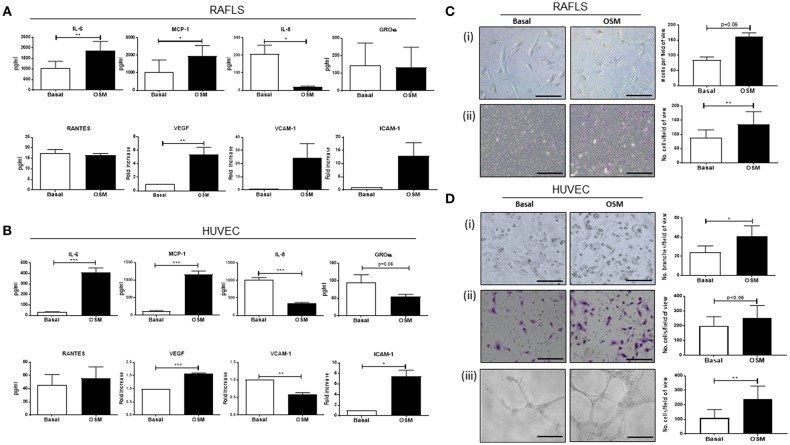
The effect of OSM on cytokine/chemokine secretion, angiogenesis, and cell function in RAFLS and HUVEC. RAFLS and HUVEC were cultured in the presence of OSM (10 ng/ml) for 24 h. **(A)** Bar graphs demonstrating quantification of IL-6, MCP-1, IL-8, GROα, RANTES secretion in RAFLS (*n* = 7–10). Gene expression analysis of VEGF, VCAM-1, and ICAM-1 quantified in RAFLS using Real-time PCR. Fold increase compared to endogenous controls (RPLPO and HPRT1) (*n* = 6–10). **(B)** Bar graphs demonstrating secretion of IL-6, MCP-1, IL-8, GROα, RANTES in HUVEC (*n* = 6–10). Gene expression analysis of VEGF, VCAM-1, and ICAM-1 quantified in HUVEC using Real-time PCR. Fold increase compared to endogenous controls (RPLPO and HPRT1) (*n* = 4). **(C)** Representative photomicrographs and accompanying bar graphs demonstrating (i) leukocyte adhesion and number of attached cells (*n* = 5), (ii) invasion and number of invading cells (*n* = 8) in RAFLS incubated with OSM for 24 and 48 h, respectively. **(D)** Representative photomicrographs and accompanying bar graphs demonstrating (i) tubule formation and average number of branches (*n* = 3), (ii) leukocyte adhesion and average number of attached cells (*n* = 6), (iii) invasion and average number of invading cells (*n* = 4) in HUVEC incubated with OSM for 24 h. Values expressed as mean ± SEM, Wilcoxon signed rank and paired *t-*test were used for RAFLS and HUVEC, respectively. *p < 0.05, **p < 0.01, ***p < 0.005 significantly different from basal.

To initially assess the effect of OSM on pro-inflammatory mechanisms, a range of pro-inflammatory mediators were measured in RAFLS and HUVEC (Figure 1). OSM significantly induced expression of IL-6, MCP-1, and ICAM-1 in RAFLS and HUVEC (all *p* < 0.05) (Figures 1A,B), in addition to the main angiogenic growth factor VEGF (*p* < 0.05) (Figures 1A,B). In contrast, OSM inhibited the secretion of IL-8 (*p* < 0.05) and GROα from both RAFLS and HUVEC (Figures 1A,B), with no effect observed for RANTES (Figures 1A,B). Interestingly, OSM induced VCAM-1 in RAFLS (Figure 1A), but inhibited VCAM-1 expression in HUVEC (*p* < 0.05) (Figure 1B). This data demonstrates the differential effects of OSM, displaying both pro-and anti-inflammatory effects in different cell types, but also within the same cell type.

### OSM Promotes Adhesion, Invasive, and Tube Formation Mechanisms in RAFLS and HUVEC

To further investigate the role of OSM we next assessed cellular function by performing adhesion, invasion and angiogenic assays. OSM stimulation significantly increased the adhesive capacity of RAFLS and HUVEC, resulting in a significant increase in PBMC attachment to the cell surface of RAFLS and HUVEC (Figures 1Ci,Di). Next, to assess the effects of OSM on RAFLS and HUVEC invasion, Transwell Matrigel™ invasion chambers were utilized. Representative images of increased RAFLS and HUVEC invasion following OSM stimulation are shown in Figures 1Cii,Dii. Quantitatively, RAFLS and HUVEC invasion were significantly induced by OSM compared to basal control (both *p* < 0.05) (Figures 1Cii,Dii). Finally, representative images of HUVEC tube formation are shown in Figure 1Diii, demonstrating a significant increase in the formation of tube-like structures in response to OSM (*p* < 0.05).

### OSM Differentially Regulates Cellular Bioenergetics in RAFLS and HUVEC

**Figure 2:**
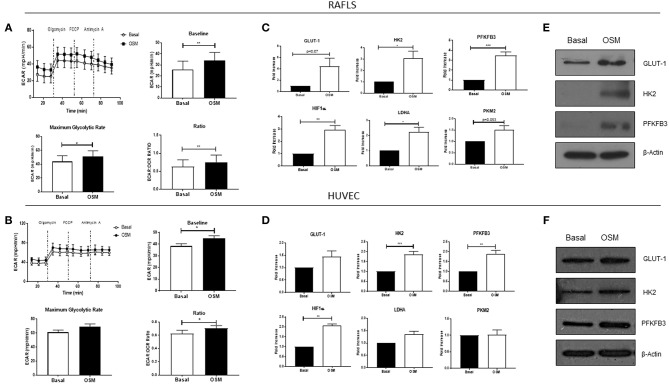
OSM induces glycolytic mechanisms in in RAFLS and HUVEC. Average seahorse profiles demonstrating extracellular acidification rate (ECAR) (glycolysis) in **(A)** RAFLS (*n* = 8) and **(B)** HUVEC (*n* = 4), before and after injections of oligomycin, FCCP, and antimycin A following 3 h OSM (10 ng/ml) stimulation. Representative bar graphs demonstrating baseline ECAR, maximal glycolytic rate and ECAR:OCR ratio in **(A)** RAFLS and **(B)** HUVEC. Representative bar graphs demonstrating mRNA expression of glucose transporter 1 (GLUT-1), hexokinase 2 (HK2), 6-phosphofructo-2-kinase/fructose-2,6-biphosphatase 3 (PFKFB3), HIF1α, lactate dehydrogenase A (LDHA glucose transporter 1 and pyruvate kinase M2 (PKM2) in **(C)** RAFLS (*n* = 5–6) and **(D)** HUVEC (*n* = 4–8) treated with OSM (10 ng/ml) for 24 h. Fold increase compared to endogenous controls (RPLPO and HPRT1). Representative western blot showing GLUT-1, HK2, PFKFB3 in **(E)** RAFLS and **(F)** HUVEC, β-actin was used as loading control. Wilcoxon signed rank and paired *t-*test were used for RAFLS and HUVEC, respectively. Data expressed as mean ± SEM, *p < 0.05, **p < 0.01, ***p < 0.005 significantly different from basal.

To analyse the two major energy pathways, oxidative phosphorylation and glycolysis, in real time the Seahorse XFe-Analyzer was utilized as previously described (29). Supplementary Figures 1A,B demonstrates the average bioenergetic profiles for OCR in RAFLS and HUVEC cells before and after injections of mitochondrial inhibitors; oligomycin, FCCP, and antimycin A in OSM vs. basal control. OSM had no effect on basal respiration in RAFLS, yet increased the maximal respiratory capacity (*p* < 0.05), paralleled by a significant reduction in ATP synthesis (*p* < 0.05) (Supplementary Figures 1A,C). OSM had no effect on the OCR profile of HUVEC (Supplementary Figures 1B,D). This was accompanied by a significant shift to a glycolytic profile of both RAFLS and HUVEC, whereby OSM significantly increased baseline glycolysis (*p* < 0.05) and maximum glycolytic capacity (*p* < 0.05), leading to an overall increase in the ECAR/OCR ratio in favor of glycolysis for both RAFLS and HUVEC (all *p* < 0.05) (Figures 2A,B). Furthermore, we demonstrated an increase in the glucose transporter GLUT-1 (Figures 2C–F) and in HIF1α (*p* < 0.01) (Figures 2C,D), a master regulator of cellular and systemic homeostatic responses to hypoxia. This glycolytic shift was further supported by the observed increase in key glycolytic enzymes HK2 (*p* < 0.05) (Figures 2C–F), the first enzyme in the glycolysis pathway, and PFKFB3 (*p* < 0.01) (Figures 2C–F), which catalyzes the conversion of fructose-6-phosphate to fructose-2,6-bisP (F2,6BP). F2,6BP is a “potent” allosteric activator of 6-phosphofructokinase-1 (PFK-1) which is one of the rate-limiting enzymes of glycolysis. LDHA and PKM2 expression were also significantly increased in RAFLS in response to OSM, with no effect observed in HUVEC (*p* < 0.05) (Figures 2C,D).

### OSM in Combination With TNFα Differentially Regulates Cytokines and Chemokines in RAFLS and HUVEC

**Figure 3:**
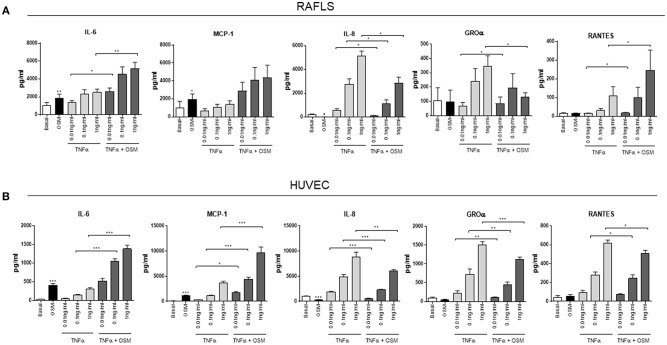
OSM in combination with TNFα regulates cytokine/chemokine secretion in RAFLS and HUVEC. RAFLS and HUVEC were cultured in the presence of OSM (10 ng/ml) and increasing concentrations of TNFα (0.01, 0.1, 1 ng/ml) for 24 h. Bar graphs showing the measured secretion of IL-6, MCP-1, IL-8, GROα, and RANTES from **(A)** RAFLS (*n* = 7–10) and **(B)** HUVEC (*n* = 10–11) following the outlined treatments by ELISA. Values expressed as mean ± SEM, Wilcoxon signed rank and paired *t-*test were used for RAFLS and HUVEC, respectively. *p < 0.05, **p < 0.01, ***p < 0.005.

We have shown that OSM displays differential effects on pro-inflammatory/angiogenic mediators in RAFLS and HUVEC, and shown that in both cell types OSM induces a shift toward glycolysis. Based on previous studies demonstrating the ability of OSM to synergise with other key cytokines within the joint environment, we next examined the effect of OSM in combination with TNFα on these mechanisms. Both RAFLS and HUVEC were cultured with increasing concentrations of TNFα, in the presence or absence of OSM. OSM potentiated the effect of TNFα on both IL-6 (*p* < 0.05) and MCP-1 (*p* < 0.05) at all concentrations in both RAFLS and HUVEC (Figures 3A,B). In contrast, OSM inhibited the stimulatory effect of TNFα on IL-8 (*p* < 0.05) and GROα (*p* < 0.05), with the levels of both chemokines significantly reduced in response to OSM + TNFα compared to TNFα alone (Figures 3A,B). Interestingly, OSM alone had no effect on RANTES secretion from both RAFLS and HUVEC, however in combination with TNFα, divergent effects were observed for RAFLS compared to HUVEC. OSM potentiated the effect of TNFα on RANTES secretion (*p* < 0.05) (Figure 3A), however OSM significantly inhibited the effect of TNFα on RANTES in HUVEC (*p* < 0.05) (Figure 3B). These data again show the divergent pro-/anti-inflammatory effects of OSM, and its ability to alter the effects of one of the main cytokines that drives inflammation within the RA joint.

### OSM in Combination With TNFα Regulates Metabolic Reprogramming in RAFLS, an Effect Mediated Through Phosphorylation of STAT3

**Figure 4:**
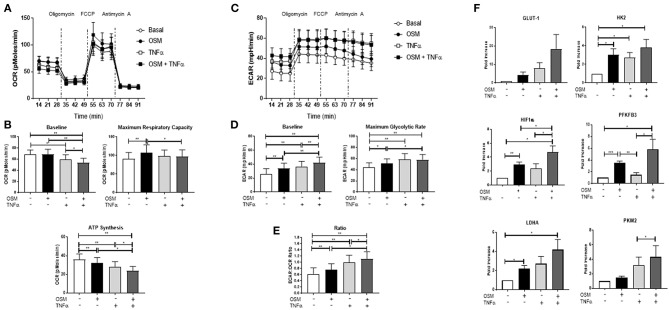
OSM in combination with TNFα regulates metabolic reprogramming in RAFLS. RAFLS were treated with OSM (10 ng/ml) alone or in combination with TNFα (1 ng/ml) for 3 h. Average seahorse profiles demonstrating **(A)** oxygen consumption rate (OCR) (oxidative phosphorylation) and **(C)** extracellular acidification rate (ECAR) (glycolysis), before and after injections of oligomycin, FCCP, and antimycin A in RAFLS (*n* = 8). Representative bar graphs demonstrating **(B)** baseline OCR, maximal respiratory capacity, ATP synthesis and **(D)** baseline OCR, maximal glycolytic rate and the ECAR:OCR ratio **(E)** (*n* = 8). **(F)** Representative bar graphs demonstrating mRNA expression of glucose transporter 1 (GLUT-1), hexokinase 2 (HK2), 6-phosphofructo-2-kinase/fructose-2,6-biphosphatase 3 (PFKFB3), HIF1α, lactate dehydrogenase A (LDHA glucose transporter 1 and pyruvate kinase M2 (PKM2) in RAFLS following treatment with OSM alone or in combination with TNFα for 24 h (*n* = 6). Fold increase compared to endogenous controls (RPLPO and HPRT1). Wilcoxon signed rank and paired *t-*test were used for RAFLS and HUVEC, respectively. Data expressed as mean ± SEM, *p < 0.05, **p < 0.01, ***p < 0.005 significantly different from basal.

To further explore the synergistic interaction between OSM and TNFα, we next examined their combined effect on cellular metabolism. While OSM had no effect on baseline OCR, TNFα alone significantly reduced baseline OCR (*p* < 0.05) (Figures 4A–C), an effect further potentiated with the combination of OSM+TNFα (*p* < 0.05) (Figures 4A–C). Maximum respiratory capacity was significantly reduced in response to the combination of OSM and TNFα (*p* < 0.05) (Figure 4B). Furthermore, the cytokines alone and in combination resulted in a stepwise inhibition of ATP synthesis (all *p* < 0.05) (Figure 4B). In contrast, OSM and TNFα alone and in combination, significantly induced a stepwise progressive increase in baseline glycolysis (all *p* < 0.05) (Figures 4C,D) and the maximal glycolytic rate (all *p* < 0.05) (Figures 4C,D). This resulted in an overall significant increase in the ECAR/OCR ratio in response to both OSM (*p* < 0.05) and TNFα (*p* < 0.05) alone, an effect that was potentiated in response to the combination (*p* < 0.05) (Figure 4E). This metabolic shift was further supported by the increased induction of GLUT-1, HK2 (*p* < 0.05), PFKFB3 (*p* < 0.05), HIF1α (*p* < 0.05), LDHA (*p* < 0.05), and PKM2 (*p* < 0.05) in response to the combination of OSM+TNFα compared to either cytokine alone (Figure 4F). In contrast, no effect was observed for baseline OCR, maximum respiratory capacity or ATP synthesis in HUVEC in response to OSM + TNFα (Supplementary Figures 2A,B). However, similar to RAFLS, OSM + TNFα induced a significant induction in the glycolytic capacity of HUVEC as demonstrated in the ECAR profiles (Supplementary Figures 2C–E) and in the expression of key glycolytic genes (Supplementary Figure 2F).

**Figure 5:**
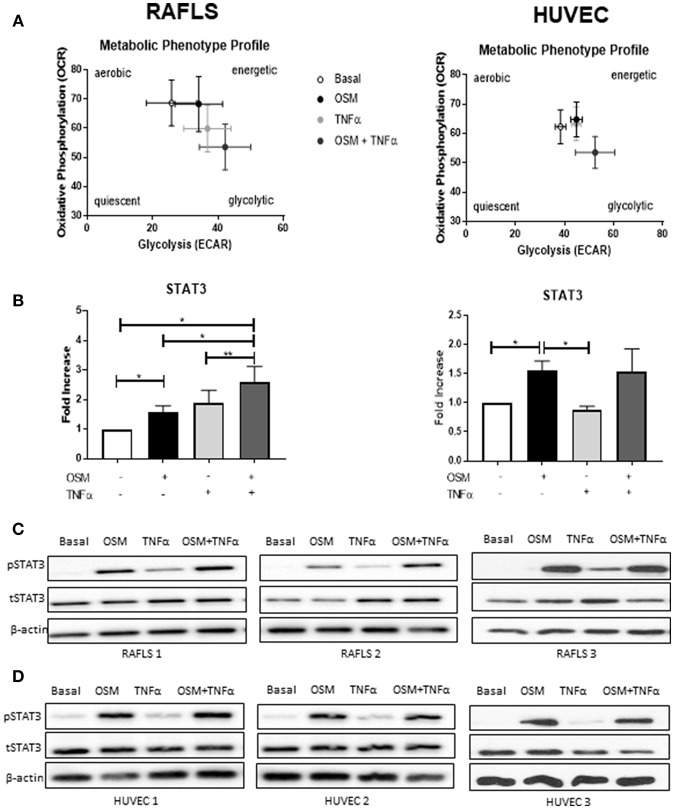
OSM in combination TNFα regulates STAT3 expression in RAFLS. **(A)** Metabolic phenotype profiles in RAFLS and HUVEC representing changes in metabolic phenotype in response to OSM (10 ng/ml) and TNFα (1 ng/ml) alone and in combination. **(B)** Representative bar graphs demonstrating mRNA expression of STAT3 in RAFLS (*n* = 6) and HUVEC (*n* = 4) following treatment with OSM alone or in combination with TNFα for 24 h. Fold increase compared to endogenous controls (RPLPO and HPRT1), data analyzed using paired *t*-test. Data expressed as mean ± SEM, *p < 0.05, **p < 0.01. Representative western blot showing phospho-STAT3 (pSTAT3) and total-STAT3 (tSTAT3) in RAFLS **(C)** and HUVEC **(D)**. β-actin was used as loading control.

The overall metabolic profile of both RAFLS and HUVEC is shown in Figure 5A, where they move toward a more glycolytic/energetic profile in response to the combination of OSM and TNFα however there are differences in the mechanisms whereby OCR was inhibited in RAFLS, with no effect observed for HUVEC. Therefore, we next assessed their effect on phosphorylation of STAT3, a key component of the JAK-STAT pathway which mediates OSM signaling. In RAFLS, OSM induced STAT3 gene expression (*p* < 0.05) (Figure 5B) and STAT3 phosphorylation (pSTAT3) as observed by western blot (Figure 5C and Supplementary Figure 3). TNFα also induced pSTAT3 but to a lesser extent (Figures 5B–D). Interestingly, the combination of OSM and TNFα in RAFLS significantly induced both gene expression compared to either OSM or TNFα alone (Figure 5B). Furthermore, the combination of OSM and TNFα in RAFLS induced activation of STAT3 (Figure 5C) in two out three RAFLS. In contrast, while OSM induced STAT3 gene expression and protein phosphorylation in HUVEC, the addition of TNFα had no effect either alone or in combination with OSM (Figures 5B,D). This suggests that in RAFLS, OSM, and TNFα have the ability to act together in the activation of STAT3, an effect that does not occur in HUVEC.

## Discussion

OSM is a crucial player in the pathogenesis of RA, however its relative contribution to specific mechanisms involved in synovial inflammation remain to be fully elucidated, primarily due to the pleiotropic nature of this cytokine. In this study we demonstrate that OSM alone differentially regulates pro-inflammatory mechanisms and significantly promotes pro-angiogenic and pro-invasive mechanisms in RAFLS and HUVEC. This is accompanied by a change in the cellular bioenergetic profile of the cells, whereby OSM significantly increases the ECAR/OCR ratio in favor of glycolysis, paralleled by the induction of the glucose transporter GLUT-1 and key glycolytic enzymes (HK2, PFKFB3, HIF1α). Next, we demonstrate that OSM synergizes with TNFα to differentially regulate pro-inflammatory mechanisms in RAFLS and HUVEC. Interestingly, OSM synergizes with TNFα to regulate metabolic reprogramming, whereby an induction of glycolytic activity with concomitant attenuation of mitochondrial respiration and ATP activity is observed in RAFLS, but not in HUVEC. Finally, we identify that the combination of OSM with TNFα induces transcriptional activity of STAT3 in RAFLS, with no effect observed in HUVEC. Together, this study indicates that OSM is an important player in orchestrating pro-inflammatory, angiogenic and invasive events in RA, specifically in RAFLS, effects that are mediated by interactions with both TNFα and STAT3.

In this study, OSM differentially regulates cytokine and chemokine secretion in both RAFLS and HUVEC, significantly inducing IL-6 and MCP-1, yet inhibiting IL-8 and GROα, with minimal effect observed on RANTES. While this is the first study to demonstrate the opposing action of OSM on these specific cytokine/chemokines in RAFLS and HUVEC, it is consistent with previous studies demonstrating differential effects in other cell types (33, 34). Specifically, OSM alone can induce GM-CSF, IL-6, growth factors VEGF and bFGF, the osteoclastogenic cytokine RANKL, and many MMP (24, 35–38). Furthermore, OSM has been shown to differentially regulate chemokines/adhesion molecules, inducing CXCL5, IP10, CCL2, MCP-1, and ICAM-1 in lung fibroblasts, osteoblasts and epithelial cells, with no effect observed for other mediators such as GROα, MIP-1, and VCAM-1 (24). The effect of OSM on chemokine expression has also been observed in mouse models of pneumonia (39), experimental autoimmune encephalomyelitis (EAE), and cancer (40, 41).

Furthermore, we demonstrate that OSM promotes pro-angiogenic mechanisms and leukocyte adhesion, accompanied by induction of VEGF and ICAM-1. VEGF is a pivotal “on” switch for angiogenesis, promoting EC proliferation, migration, and invasion (42–45), with numerous studies demonstrating increased expression of VEGF and its receptors in RA synovial tissue (42, 46, 47). This is further supported by studies indicating that OSM can have differential effects on angiogenic mechanisms dependent on STAT activation, with pSTAT1 inhibiting VEGF expression, yet pSTAT3 promoting VEGF expression (48, 49). The effect of OSM on leukocyte adhesion has also been observed in mouse models of arthritis (50). OSM has also been shown to upregulate the expression of CCL13 in RAFLS (51), to induce key chemokines involved in leukocyte chemotaxis (CXCL3, CCL2, CCL5, CCL20), in addition to promoting infiltration of macrophages and neutrophils in mice models of inflammation (52). The observed differential effects of OSM on VCAM-1 expression again highlights its pleiotropic nature in different cell types, possibly influenced by the inflammatory microenvironment.

These striking changes in cellular function are, in fact, mirrored by distinct alterations in the metabolic profiles of OSM-treated cells, resulting in a shift in the ECAR:OCR ratio in favor of glycolysis. This shift is supported by the observed increase in key glycolytic drivers in response to OSM treatment, where induction of HIF1α, PFKFB3, HK2, LDHA, PKM2, and GLUT-1 was demonstrated. The metabolic switch in HUVEC in response to OSM is consistent with previous studies indicating that active endothelial cells rely heavily on glycolysis. Indeed, 85% of endothelial cell ATP requirements comes from the conversion of glucose to lactate, mechanisms that are crucial for tip cell formation and blood vessel migration (53–55). This preferential use of glycolysis has also been demonstrated in the inflamed RA joint, with studies showing that glycolytic markers are inversely correlated with synovial pO_2_ levels (9, 56, 57). In addition, previous studies have shown that OSM can promote glycolytic mechanisms in human hepatocyte cell lines in a PDK-1-dependent manner and can induce HIF1α in different cell types to promote tumor progression in cancer cells (58, 59). Consistent with the observed increase in PFKFB3 in this study, previous studies have reported that blockade of PFKFB3 inhibits angiogenic tube formation, secretion of pro-inflammatory/angiogenic mediators, and key signaling pathways in both RAFLS and endothelial cells (9). Moreover, blockade of PFKFB3 in animal models of RA, psoriasis and colitis has led to resolution of inflammation (60, 61).

The inflamed synovial joint is hallmarked by a complex mixture of pro-inflammatory cytokines and chemokines interacting with each other to promote the inflammatory response. In this study we demonstrate that OSM potentiates the effect of TNFα on IL-6 and MCP-1 secretion from HUVEC and RAFLS, inhibits TNFα-induced IL-8 and GROα, while displaying differential effects on RANTES, with OSM significantly inhibiting TNFα-induced RANTES expression in HUVEC, while potentiating TNFα-induced RANTES in RAFLS. The ability of OSM to cooperate with key pro-inflammatory mediators such as IL-1β, IL-17, and TNFα has been previously reported (25, 31, 62). In mouse synovial fibroblasts, OSM augments the effects of TNFα and IL-1β on IL-6 secretion (38), inhibits IL-1β-induced IL-8 and GM-CSF expression (30), and can synergise with TLR-4 to induce MCP-1 in human aortic adventitious fibroblasts and smooth muscle cells (63). Furthermore, OSM inhibits TNFα-induced TIMP-1 expression, yet potentiates IL-1β-induced TIMP-1 and MMP-1 in RAFLS (24, 31, 64).

Interestingly, we identified that the synergistic effects observed with OSM and TNFα together also differentially altered the metabolic profile of the cells. Specifically, the combination of OSM and TNFα reduced the mitochondrial respiration paralleled by a stepwise induction of glycolysis in RAFLS, an effect not observed in HUVEC. Indeed, we demonstrate that the synergy between OSM and TNFα observed in RAFLS may be STAT3-dependent, an effect that appears to be specific to RAFLS and not HUVEC. In line with this, studies have demonstrated that TNFα is capable of indirectly activating the JAK-STAT pathway through induction of type I interferons in RAFLS (65). The mechanisms by which OSM regulates such effects within the inflamed joint however is unclear, yet studies have suggested that differential activation of its receptors gp130/LIFα and gp130/OSM or differential combinations of STATs (whether they form hetero- or homo-dimers) may account for such opposing effects (16, 66).

Furthermore, OSM has been shown to regulate STAT1/3 and STAT5/6 in mouse fibroblasts and is also capable of suppressing cell motility via STAT1 activation in lung cancer (67). Conversely, a recent study has demonstrated that murine OSM phosphorylates STAT3 via gp130/LIF activation but not STAT1 causing specific regulation of STAT3 responsive genes in primary osteocytes (68). Indeed, STAT3 itself is capable of interacting with other STATs; STAT1 for example has been demonstrated to exhibit inhibitory effects against STAT3 signaling in a study on esophageal squamous cell carcinoma (69). Thus, a clearer understanding of the various cues directing this complex transcriptional landscape is vitally important.

In this study, we propose that the altered cellular bioenergetics resulting from the synergy between OSM and TNFα may rely on STAT3 activation in RAFLS. Interactions between STAT3 and metabolic enzymes have also been demonstrated previously whereby blocking PFKFB3 causes inhibition of pSTAT3 expression in RAFLS (9). In cancer cells, STAT3 regulates glycolysis through HK2 (70, 71), and mediates HIF1α-PKM2-interactions (54). Furthermore, STAT3, has been shown to be localized in the mitochondria, can bind to complex I and, in liver and heart cells, is capable of modulating the electron transport chain by altering activities of complex I and II (72). Finally, in the context of the RA joint, STAT3 interacts with various other key signaling molecules including Notch, NF-κB, and hypoxia inducible factors (HIF), all of which regulate each other's activation through complex positive and negative feedback loops in the RA joint (73).

In conclusion, we have shown that OSM is capable of driving pro-inflammatory and metabolic changes, implicating it as a crucial cytokine in orchestrating the inflammatory response in rheumatoid arthritis. Moreover, we demonstrate that OSM enhances the destructive effects of TNFα, a key pathogenic factor in disease pathogenesis, effects which are mediated through activation of STAT3.

## Data Availability

All datasets generated for this study are included in the manuscript/Supplementary Files.

## Author Contributions

MH designed and performed experiments, analyzed data, and wrote the manuscript. TR, CC, and SA performed experiments. DV recruited the patients and contributed to the discussion. TM and UF supervised the project and co-wrote the manuscript. All authors read, revised, and approved the submitted manuscript.

### Conflict of Interest Statement

The authors declare that the research was conducted in the absence of any commercial or financial relationships that could be construed as a potential conflict of interest.

## Supplementary Material

Supplementary Figure 1 OSM has differential effects on mitochondrial respiration in RAFLS and HUVEC. Average seahorse profiles demonstrating oxygen consumption rate (OCR) (oxidative phosphorylation) in **(A)** RAFLS (*n* = 8) and **(B)** HUVEC (*n* = 4), before and after injections of oligomycin, FCCP, and antimycin A following 3 h OSM (10 ng/ml) stimulation. **(C)** Representative bar graphs demonstrating baseline OCR, maximal respiratory capacity, and ATP synthesis in RAFLS (*n* = 8). **(D)** Representative bar graphs demonstrating baseline OCR, maximal respiratory capacity, and ATP synthesis in HUVEC (*n* = 4). Wilcoxon signed rank and paired *t-*test were used for RAFLS and HUVEC, respectively. Data is expressed as mean ± SEM, ^**^*p* < 0.01 significantly different from basal.

Supplementary Figure 2 OSM does not synergise with TNFα to regulate metabolic changes in HUVEC. HUVEC were treated with OSM (10 ng/ml) alone or in combination with TNFα (1 ng/ml) for 3 h. Average seahorse profiles demonstrating **(A)** oxygen consumption rate (OCR) (oxidative phosphorylation) and **(C)** extracellular acidification rate (ECAR) (glycolysis), before and after injections of oligomycin, FCCP, and antimycin A (*n* = 4). Representative bar graphs demonstrating **(B)** baseline OCR, maximum respiratory capacity, ATP synthesis and **(D)** baseline ECAR, maximal glycolytic rate and **(E)** ECAR:OCR ratio. **(F)** Representative bar graphs demonstrating mRNA expression of glucose transporter 1 (GLUT-1), hexokinase 2 (HK2), 6-phosphofructo-2-kinase/fructose-2,6-biphosphatase 3 (PFKFB3), HIF1α, lactate dehydrogenase A LDHA glucose transporter 1 and pyruvate kinase M2 (PKM2) in HUVEC treated with OSM alone or in combination with TNFα for 24 h (*n* = 4–5). Fold increase compared to endogenous controls (RPLPO and HPRT1). Data analyzed using paired *t-*test, data expressed as mean ± SEM, ^*^*p* < 0.05, ^**^*p* < 0.01, ^***^*p* < 0.005 significantly different from basal.

Supplementary Figure 3 OSM in combination TNFα regulates STAT3 expression. Bar graphs representing densitometry quantification of pSTAT3 normalized to β-actin in RAFLS **(A)** and HUVEC **(B)**. Data expressed as mean ± SEM.
