# Supplementary figures and images for: STAT3 Mediates the Differential Effects of Oncostatin M and TNFα on RA Synovial Fibroblast and Endothelial Cell Function
Source: Front Immunol. 2019 Aug 28;10:2056. doi: 10.3389/fimmu.2019.02056 (PMC6724663; doi:10.3389/fimmu.2019.02056)

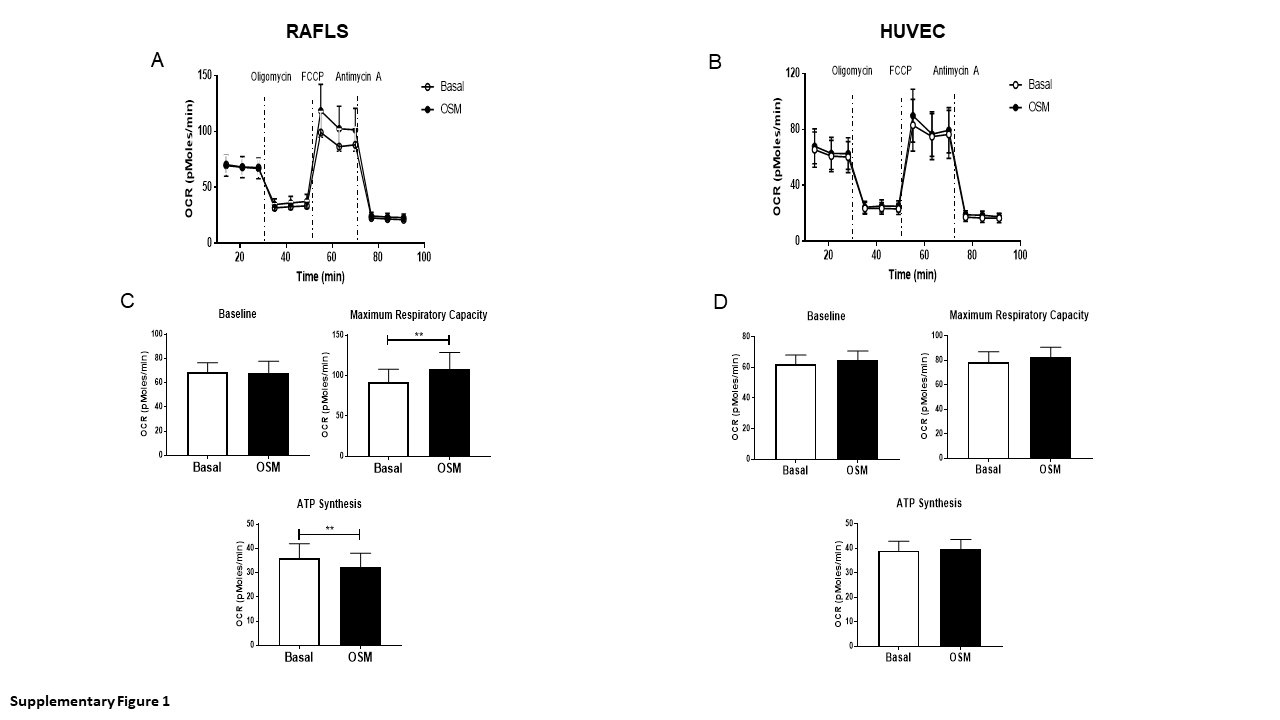

Supplement: Supplementary Figure 1 — OSM has differential effects on mitochondrial respiration in RAFLS and HUVEC. Average seahorse profiles demonstrating oxygen consumption rate (OCR) (oxidative phosphorylation) in (A) RAFLS (n = 8) and (B) HUVEC (n = 4), before and after injections of oligomycin, FCCP, and antimycin A following 3 h OSM (10 ng/ml) stimulation. (C) Representative bar graphs demonstrating baseline OCR, maximal respiratory capacity, and ATP synthesis in RAFLS (n = 8). (D) Representative bar graphs demonstrating baseline OCR, maximal respiratory capacity, and ATP synthesis in HUVEC (n = 4). Wilcoxon signed rank and paired t-test were used for RAFLS and HUVEC, respectively. Data is expressed as mean ± SEM, **p < 0.01 significantly different from basal. [file Image_1.tif]

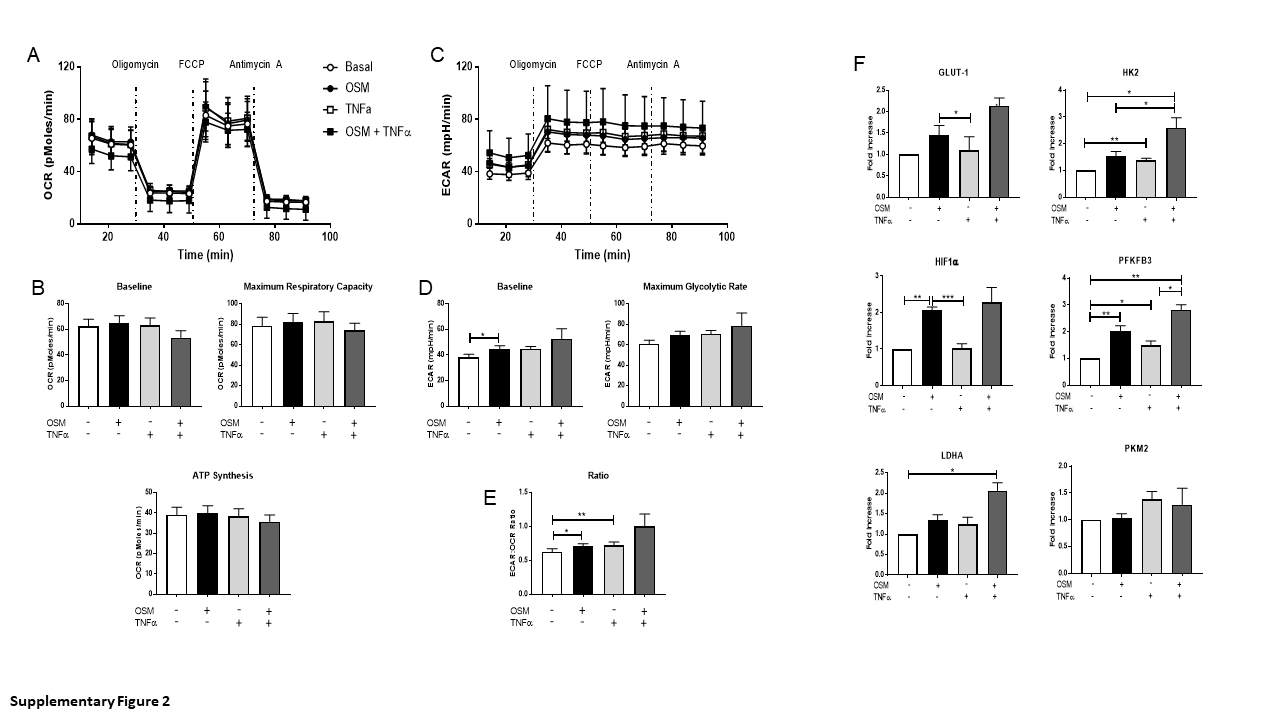

Supplement: Supplementary Figure 2 — OSM does not synergise with TNFα to regulate metabolic changes in HUVEC. HUVEC were treated with OSM (10 ng/ml) alone or in combination with TNFα (1 ng/ml) for 3 h. Average seahorse profiles demonstrating (A) oxygen consumption rate (OCR) (oxidative phosphorylation) and (C) extracellular acidification rate (ECAR) (glycolysis), before and after injections of oligomycin, FCCP, and antimycin A (n = 4). Representative bar graphs demonstrating (B) baseline OCR, maximum respiratory capacity, ATP synthesis and (D) baseline ECAR, maximal glycolytic rate and (E) ECAR:OCR ratio. (F) Representative bar graphs demonstrating mRNA expression of glucose transporter 1 (GLUT-1), hexokinase 2 (HK2), 6-phosphofructo-2-kinase/fructose-2,6-biphosphatase 3 (PFKFB3), HIF1α, lactate dehydrogenase A LDHA glucose transporter 1 and pyruvate kinase M2 (PKM2) in HUVEC treated with OSM alone or in combination with TNFα for 24 h (n = 4–5). Fold increase compared to endogenous controls (RPLPO and HPRT1). Data analyzed using paired t-test, data expressed as mean ± SEM, *p < 0.05, **p < 0.01, ***p < 0.005 significantly different from basal. [file Image_2.tif]

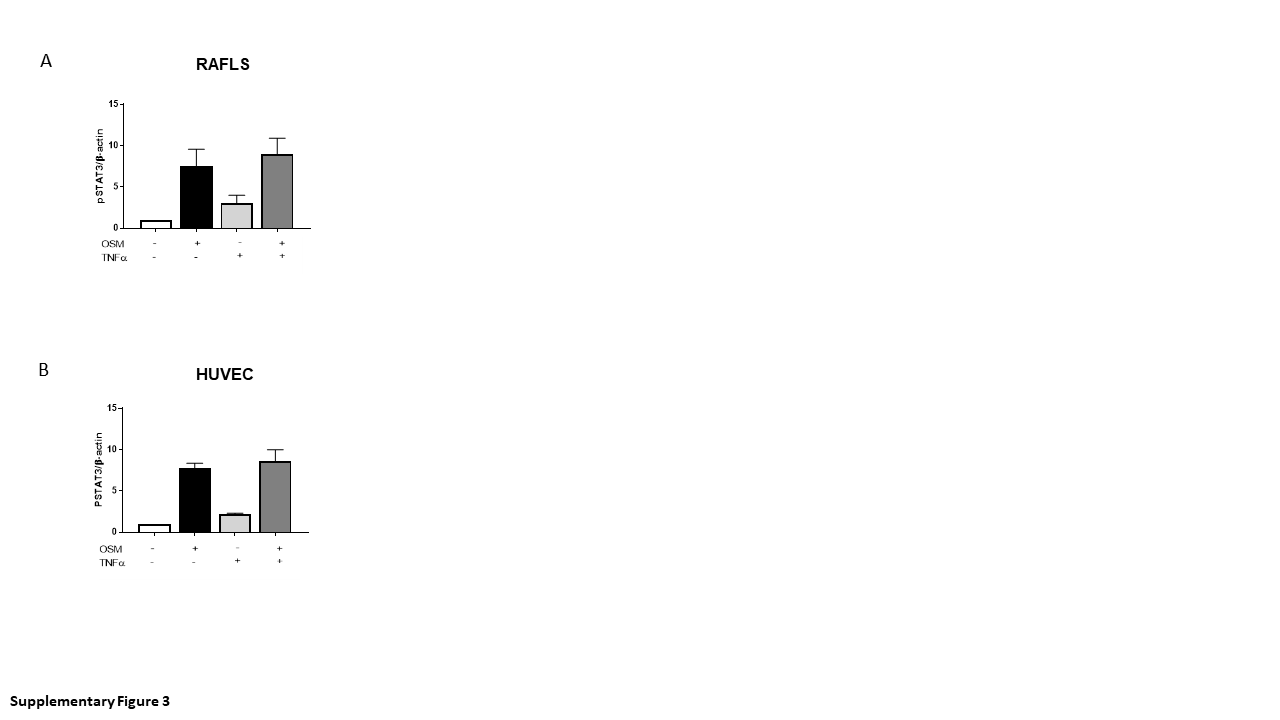

Supplement: Supplementary Figure 3 — OSM in combination TNFα regulates STAT3 expression. Bar graphs representing densitometry quantification of pSTAT3 normalized to β-actin in RAFLS (A) and HUVEC (B). Data expressed as mean ± SEM. [file Image_3.tif]
